# 1,6-Hexanediol regulates angiogenesis via suppression of cyclin A1-mediated endothelial function

**DOI:** 10.1186/s12915-023-01580-8

**Published:** 2023-04-07

**Authors:** Yongying Jiang, Gongyun Lei, Ting Lin, Nan Zhou, Jintao Wu, Zhou Wang, Yihui Fan, Hongzhuan Sheng, Renfang Mao

**Affiliations:** 1 Department of Pathophysiology, School of Medicine, Nantong University, 19 Qixiu Road, Nantong, Jiangsu 226001 People’s Republic of China; 2 Heart Center of Henan Provincial People’s Hospital, Central China Fuwai Hospital, Central China Fuwai Hospital of Zhengzhou University, Zhengzhou, Henan 450003 People’s Republic of China; 3 Laboratory of Medical Science, School of Medicine, Nantong University, Nantong, Jiangsu 226001 People’s Republic of China; 4 Department of Pathogenic Biology, School of Medicine, Nantong University, Nantong, Jiangsu 226001 People’s Republic of China; 5 Department of Cardiology, Affiliated Hospital of Nantong University, Nantong University, 19 Qixiu Road, Nantong, Jiangsu 226001 People’s Republic of China

**Keywords:** Angiogenesis, Endothelial cells, LLPS, 1,6-HD, Cyclin A1

## Abstract

**Background:**

Angiogenesis plays important roles in physiological and pathologic conditions, but the mechanisms underlying this complex process often remain to be elucidated. In recent years, liquid–liquid phase separation (LLPS) has emerged as a new concept to explain many cellular functions and diseases. However, whether LLPS is involved in angiogenesis has not been studied until now. Here, we investigated the potential role of LLPS in angiogenesis and endothelial function.

**Results:**

We found 1,6-hexanediol (1,6-HD), an inhibitor of LLPS, but not 2,5-hexanediol (2,5-HD) dramatically decreases neovascularization of Matrigel plug and angiogenesis response of murine corneal in vivo. Moreover, 1,6-HD but not 2,5-HD inhibits microvessel outgrowth of aortic ring and endothelial network formation. The endothelial function of migration, proliferation, and cell growth is suppressed by 1,6-HD. Global transcriptional analysis by RNA-sequencing reveals that 1,6-HD specifically blocks cell cycle and downregulates cell cycle-related genes including cyclin A1. Further experimental data show that 1,6-HD treatment greatly reduces the expression of cyclin A1 but with minimal effect on cyclin D1, cyclin E1, CDK2, and CDK4. The inhibitory effect of 1,6-HD on cyclin A1 is mainly through transcriptional regulation because proteasome inhibitors fail to rescue its expression. Furthermore, overexpression of cyclin A1 in HUVECs largely rescues the dysregulated tube formation upon 1,6-HD treatment.

**Conclusions:**

Our data reveal a critical role of LLPS inhibitor 1,6-HD in angiogenesis and endothelial function, which specifically affects endothelial G1/S transition through transcriptional suppression of *CCNA1*, implying LLPS as a possible novel player to modulate angiogenesis, and thus, it might represent an interesting therapeutic target to be investigated in clinic angiogenesis-related diseases in future.

**Supplementary Information:**

The online version contains supplementary material available at 10.1186/s12915-023-01580-8.

## Background

Angiogenesis, a process of blood vessel formation from pre-existing vascular structures, has a pivotal impact on human health and the pathological process of many diseases, such as female menstrual cycle, tissue growth, wound healing, cancer, ischemia, inflammatory diseases, and blinding eye diseases [1]. The process of angiogenesis is extremely complicated and dynamic, which includes endothelial cells transition from resting to activated state, new capillary tube formation, vascular elongation, further sprouting, and remodeling [2, 3]. Angiogenesis is triggered by growth factors and tightly regulated by signaling pathways [4, 5]. In the whole orchestrated process, although several components, for example, growth factor interactions, vascular pericytes, basement membrane, and the extracellular matrix are involved, endothelial cells (EC) are considered as the main players in angiogenesis. EC proliferation, migration, sprouting, branching, and tube formation are the key steps of angiogenesis. Inhibition of angiogenesis is believed as an ideal strategy for the treatment of excessive angiogenesis-related diseases. However, currently available antiangiogenic therapies, even growth factor-based (e.g., anti-VEGF [vascular endothelial growth factor]), show limited efficacy [6]. In recent years, despite great progress has been made in identifying the molecular pathways and endothelial cell metabolism that modulate angiogenesis, our understanding of this process is still incomplete. Therefore, it is necessary to have new concept to overcome this limitation to further comprehend the complicated mechanisms of angiogenesis.

Liquid–liquid phase separation (LLPS) is a reversible and dynamic process by formation of weak and multivalent interactions between biomolecules, which attracts scientist’s extensive attention, and is believed as a new concept to understand the mechanisms of some complex diseases recently. It was used to explain the problem with the different non-membrane-bound compartments coexisting in cytoplasm at the very beginning [7]. The non-membrane-bound compartment is theorized to be in a liquid-like state or drop, in which the components can rearrange easily [8]. Along with the in-depth of the research, LLPS is found to be involved in several fundamental cellular functions. Current experiment evidence has shown that it plays important roles in nuclear pore passage [9], heterochromatin formation [10, 11], nucleoli formation [12], mitosis [13], intracellular signal transduction [14, 15], and extracellular signal response [16]. Moreover, it is proposed to be associated with numerous diseases, including neurodegenerative disease, cancer, and infectious diseases [17]. Although more experiment evidence needs to be performed, LLPS is thought as a new principle to interpret lots of diseases, many intracellular functions, and some extracellular activities [18]. The critical function of LLPS in physiological and pathological process has been recognized ongoing. Angiogenesis is regulated by endothelial cellular and extracellular activities and substantially contributes to not only our physiological health condition but also pathogenesis of many diseases settings, and thus, we propose that LLPS as a new concept might be involved in angiogenesis and endothelial function. To this end, we for the first time investigated the possibility of LLPS involved in angiogenesis.

To assess the potential role of LLPS in angiogenesis, we employed 1,6-hexanediol (1,6-HD), which is considered as an inhibitor of LLPS and a powerful tool to study the physical function of membrane-less compartments. 1,6-HD, an aliphatic alcohol, specifically disrupts multivalent hydrophobic interactions between protein-RNA or protein–protein that are favorable to the formation of LLPS [10, 19, 20]. It has been reported that 1,6-HD affects cell viability in HeLa cells while the cytotoxicity of 1,6-HD on EA.hy926 cells is minimal [21, 22]. Therefore, it is plausible to reason 1,6-HD may have function in endothelial cells. But the direct experiment study of 1,6-HD in the regulation of endothelial function and blood vessel formation and in the control of the process of angiogenesis still remain to be explored. In the present study, we systemic examined the role of 1,6-HD in major aspects of endothelial function such as proliferation, migration, network formation and cell cycle, and angiogenesis by performing in vivo corneal micropocket, Matrigel plug assay, and aortic ring assay. Our results showed that 1,6-HD impairs angiogenesis and endothelial function. By further screening and identifying the molecular of the biological effect of 1,6-HD on EC function and angiogenesis, cyclin A1 was validated as the molecular target of 1,6-HD in EC. Overexpressed cyclinA1 in EC could rescue inhibitory effect on tube formation by 1,6-HD. Besides, we used 2,5-hexanediol (2,5-HD) which is also an aliphatic alcohol but has no effect on phase separated in living cells [23]. Interestingly, 2,5-hexanediol fails to affect tube formation in HUVECs, aortic ring, and in vivo angiogenesis assays. Our findings uncovered an important role of LLPS inhibitor 1,6-HD in endothelial function and angiogenesis, suggesting that LLPS is a potential novel player in the regulation of angiogenesis.

## Results

### 1,6-Hexanediol but not 2,5-hexanediol robustly reduced pathological angiogenesis in mice

**Fig. 1:**
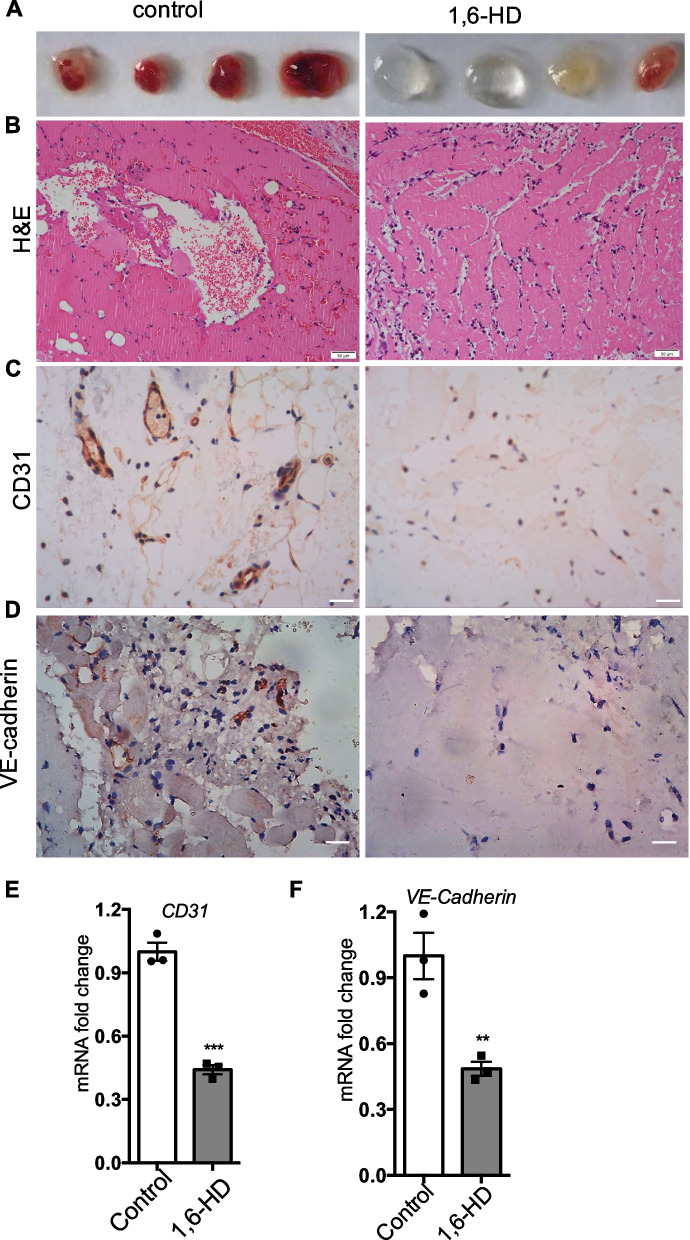
1,6-Hexanediol regulates neovascular formation in Matrigel plugs. **A** The gross morphology of Matrigel plugs was shown. **B**–**D** The hematoxylin and eosin (H&E) staining, CD31, and VE-cadherin immunohistochemistry of Matrigel plugs were performed and shown. **E**, **F** Levels of CD31 and VE-cadherin were determined by quantitative real-time polymerase chain reaction. Mean viability was shown and standard error of the mean presented the standard deviations of triplicate samples. **p < 0.01, ***p < 0.001. Scale bar: 50 μm

To access whether liquid–liquid phase separation might play a role in pathological angiogenesis, we used 1,6-hexanediol, an inhibitor of LLPS, which mixed into Matrigel to carry out in vivo pathological angiogenesis assay. Matrigel mixed without or with 1,6-HD was injected into C57BL/6 mice subcutaneously. The liquid Matrigel solidifies to a form of plug when injected into mice at the body temperature. This is a widely used an in vivo assay to evaluate angiogenesis. Five days later after injection, the Matrigel plugs were isolated. H&E staining and immunohistochemistry were then performed to determine the formation of neovasculature. Interestingly, the data showed that Matrigel plugs containing 1,6-HD robustly inhibits blood vessel formation, while the plugs from control group showed a proper vessels imbuement from bright field images, H&E, CD31, and VE-cadherin immunohistochemistry (IHC) staining (Fig. 1A–D). We then did quantitative RT-PCR analysis and found the expressions of endothelial cell markers CD31 and VE-cadherin are significantly decreased in Matrigel plugs from mixed with 1,6-HD (Fig. 1E and F). Our data suggest that 1,6-HD inhibits pathological angiogenesis.

1,6-HD inhibits LLPS by specifically disrupting weak hydrophobic interactions [10]. Like 1,6-HD, 2,5-hexanediol (2,5-HD) is also an aliphatic alcohol but does not affect LLPS in living cells [23]. Accordingly, we examined whether 2,5-HD has an effect on angiogenesis in Matrigel plug assay. Excitingly, the data from bright field, H&E, and IHC staining showed that 2,5-HD has no apparent effect on neovascular formation in Matrigel (Figure S1A-D). Therefore, these data indicate that 1,6-HD but not 2,5-HD limits pathological angiogenesis and liquid–liquid phase separation might be involved in angiogenesis.

**Fig. 2:**
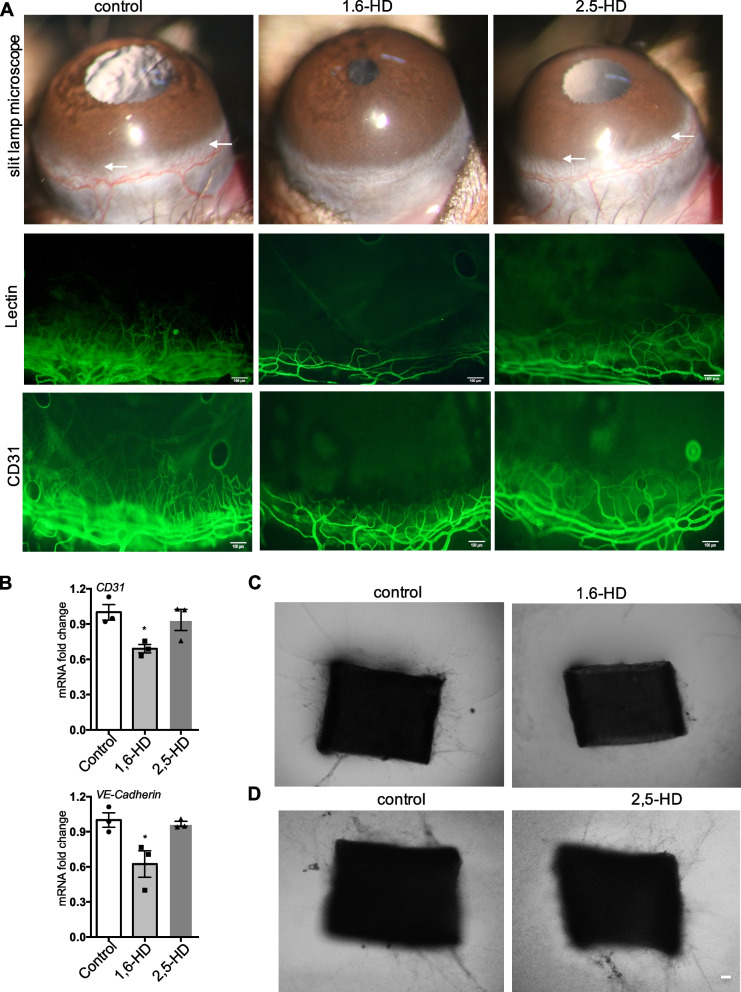
1,6-Hexanediol but not 2,5-hexanediol impairs corneal angiogenesis and microvessel outgrowth of aortic ring. **A** Slip lamp microscope images and immunofluorescence of FITC-lectin and CD31 were shown. **B** Levels of CD31 and VE-cadherin mRNA expression were shown. **C**, **D** Representative images of the capillary sprouts from the aorta rings. Scale bar: 100 μm (**A**), 200 μm (**C**)

To further determine the potential role of LLPS in pathological angiogenesis, we employed another well-established in vivo angiogenesis model, corneal angiogenesis assay, which is easy to monitor the neovascularization by a slit lamp. Micropelletes containing 1,6-HD/2,5-HD or vehicle control were implanted in micropockets in the eyes of C57BL/6 mice. Corneal angiogenesis response was evaluated under slit lamp microscope after 7 days’ implantation. The corneal immunofluorescence of FITC-lectin and CD31was further performed to evaluate the blood vessel formation. Mice with 1,6-HD display greatly decreased blood vessel growth compared with those control mice, while 2,5-HD fails to do that (Fig. 2A). The expression of CD31 and VE-cadherin in cornea is significantly reduced in 1,6-HD but not 2,5-HD group (Fig. 2B). This result confirmed the inhibited effect of 1,6-HD but not 2,5-HD on pathological angiogenesis. Collectively, these data demonstrated that 1,6-HD inhibits angiogenesis, indicating LLPS might be involved in angiogenesis.

### 1,6-Hexanediol, but not 2,5-hexanediol impaired microvessel outgrowth of aortic ring and network formation of endothelial cells

We next performed aortic ring assay using 2,5-HD and 1,6-HD treatment. 1,6-HD stimulated aortic ring considerably suppresses microvessel outgrowth, while the outgrowth is clearly visible in control (Fig. 2C). By contrast, 2,5-HD does not significantly affect microvessel outgrowth even in the presence of 20 mg/ml (Fig. 2D). It suggests 1,6-HD inhibits endothelial cell function and further supports a role of LLPS in angiogenesis.

**Fig. 3:**
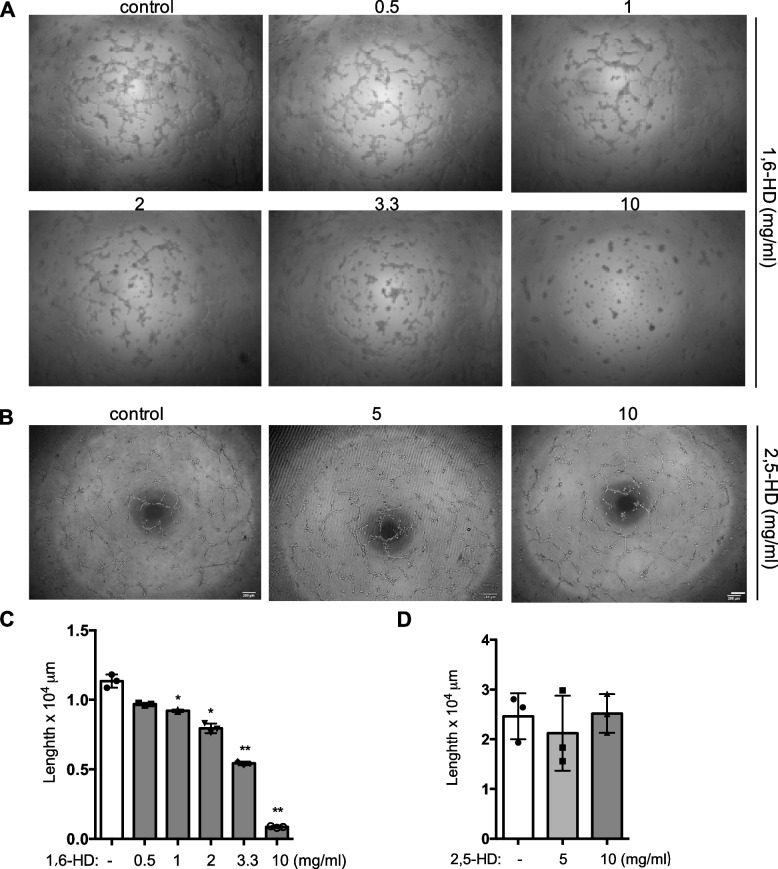
1,6-Hexanediol but not 2,5-hexanediol inhibits tube formation of endothelial cell. **A**, **B** Tube formation assay was performed. Micrographs of network formation was taken. **C**, **D** Quantification of **A** and **B**. Mean viability was shown and standard error of the mean presented the standard deviations of triplicate samples. *p < 0.05, **p < 0.01. Scale bar: 200 μm

To substantiate our above in vivo findings, we sought to perform in vitro angiogenesis assay to investigate the effect of LLPS on endothelial cell function in human umbilical vein endothelial cells (HUVECs), since 1,6-HD has been reported to affect cell viability in HeLa cells [21] but maintains cell viability in EA.hy926 cells [22]. We firstly examined the cytotoxicity of 1,6-HD in HUVECs by 1,6-HD in a wide range of doses. Consistent with previous studies in EA.hy926, our data in HUVECs exhibits insignificant cytotoxic effect in the relative low concentrations, but the cytotoxic effect increases with the increases of concentrations (Fig. S2 and S3). It suggested that endothelial cell is relatively resistant to 1,6-HD compared the reported HeLa cells. We then did tube formation and the data showed that endothelial network formation is inhibited by 1,6-HD treatment with a dose-dependent manner (Fig. 3A and C). No significant difference of tube formation was found between 2,5-HD and its control treatments (Fig. 3B and D). Taken together, these data strongly indicate that LLPS might have a role in angiogenesis.

### 1,6-Hexanediol suppresses migration, proliferation, and cell growth of endothelial cells

**Fig. 4:**
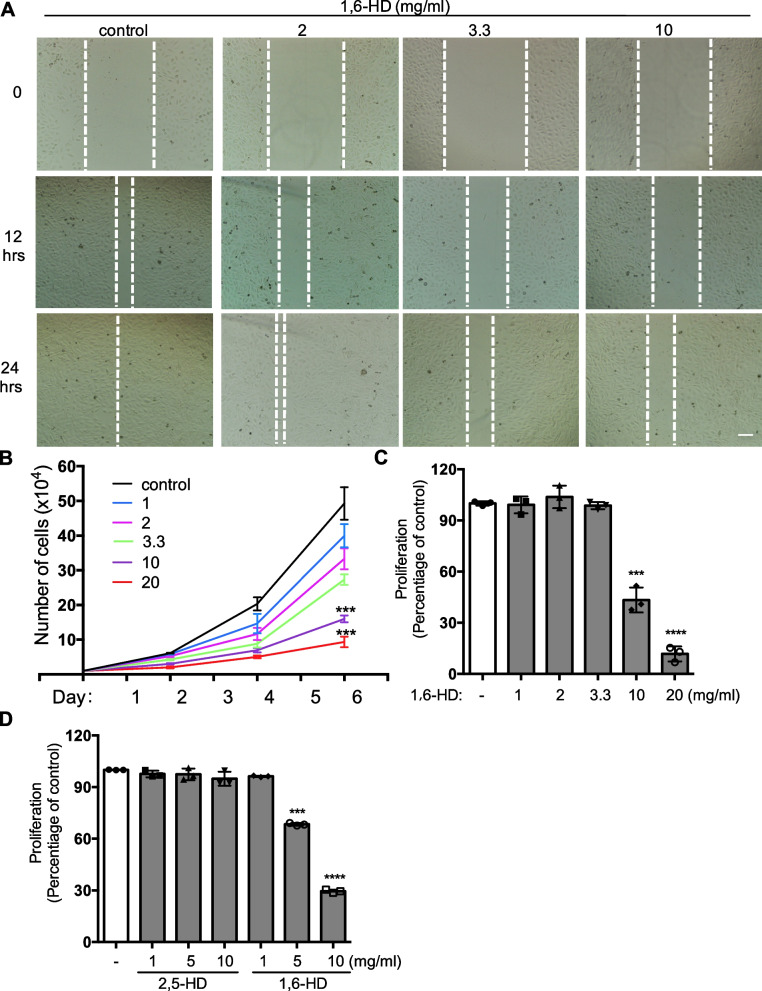
1,6-Hexanediol affects the endothelial function. **A** HUVECs were treated by 1,6-HD at indicated concentrations for 16 h. Cell migration was observed by scratch healing assay at 12 h and 24 h after scratching. **B** Cell growth curve was recorded in HUVECs with or without indicated concentrations of 1,6-HD for 6 days by counted every other day. **C**, **D** Cell proliferation was determined by CCK8 assay in HUVECs in the presences of the indicated concentration of 1,6-HD or 2,5-HD for 16 h. Mean viability was shown and standard error of the mean presented the standard deviations of triplicate samples. ***p < 0.001, ****p < 0.0001. Scale bar: 100 μm

To further examine the effect of 1,6-HD on endothelial function, we checked the cell migration, cell growth and proliferation in HUVECs in the treatment with 1,6-HD. HUVECs were placed in a 6-well plate before a pipette tip to scratch and then cultured with low fetal bovine serum condition. Twelve and twenty-four hours later, compared to control cells, cells treated by 1,6-HD showed an impaired scratch healing ability with a dose-dependent manner (Fig. 4A). This result prompted us to study the effect of 1,6-HD on endothelial cell growth. HUVECs were treated with the indicated concentrations of 1,6-HD. Total cell numbers were counted every other day for 6 days. The data showed that cell growth is also attenuated under the treatment of 1,6-HD (Fig. 4B). Next, we assessed whether 1,6-HD affects the proliferation of endothelial cells by CCK8 assay. As shown in Fig. 4C, 1,6-HD significantly decreases the proliferation ability of HUVECs. These results suggested that the inhibitory effect of 1,6-HD on HUVECs is likely due to an impairment of endothelial cell proliferation.

Because we found that 1,6-HD but not 2,5-HD controls microvessel outgrowth and network formation, we then checked the effects of 2,5-HD on the proliferation of HUVECs to further verify it. Three representative doses were chosen to use of 1,6-HD and 2,5-HD respectively. As expected, 2,5-HD failed to impair the proliferation of HUVECs, while 1,6-HD also showed an inhibitory effect on the proliferation of HUVECs (Fig. 4D), which further supports a role of LLPS in angiogenesis and endothelial function.

### 1,6-Hexanediol regulates endothelial cell cycle

**Fig. 5:**
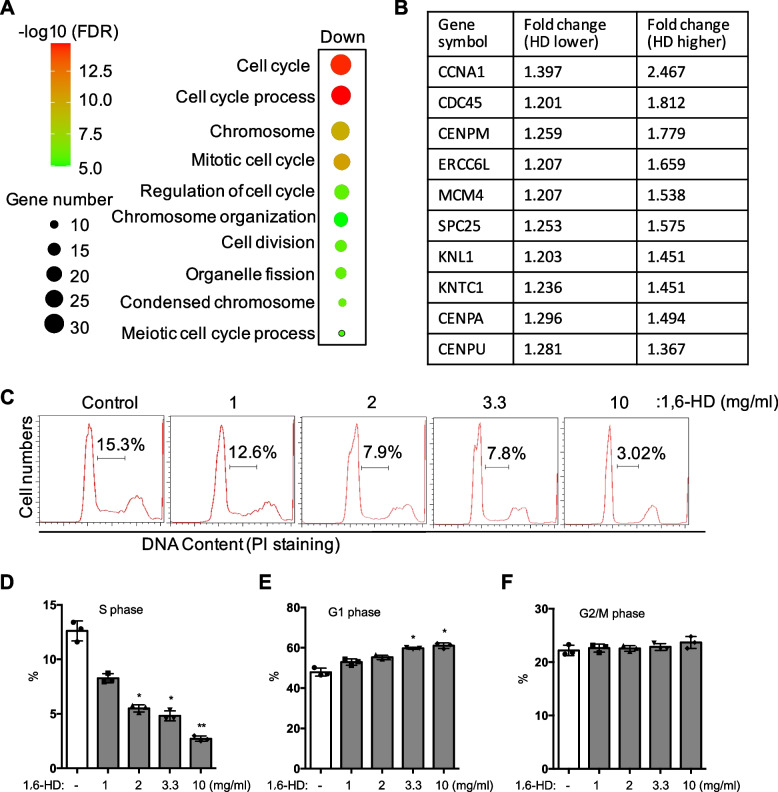
1,6-Hexanediol affects endothelial cell cycle. **A**, **B** HUVECs were treated by two doses of 1,6-HD. HD lower: 1 mg/ml, HD higher: 3.3 mg/ml. Cells were sent to Beijing Genomic Institution (BGI) for RNA sequencing analysis. The most downregulated genes in 1,6-HD treated cells were analyzed by online gene set enrichment analysis (GSEA) program. The most changed top 10 cell cycle genes with dose-dependent manner after 1,6-HD treatment were listed. **C** HUVECs were treated with 1,6-HD at indicated concentrations. Cell cycle was analyzed by flow cytometry. **D**–**F** The percentages of S phase, G1 phase, and G2/M phase were further analyzed. Mean viability was shown and standard error of the mean presented the standard deviations of duplicated samples. *p < 0.05, **p < 0.01

Based on the above results that 1,6-HD regulates angiogenesis and endothelial cell migration, proliferation, and cell growth, we next focused on how 1,6-HD plays a role in it. To address this, we carried out RNA sequencing analysis after 1,6-HD treatment in HUVECs. We used two dose of 1,6-HD 1 and 3.3 mg/ml which defined as lower and higher. One thousand three hundred four genes are differentially expressed between control and treated group. Further analysis based on fold of change and level of expression, most affected genes including 91 upregulated genes and 115 downregulated genes are dose dependent and further analyzed. By gene set enrichment analysis, the result of downregulated genes is significantly enriched in cell cycle (Fig. 5A). In contrast, the upregulated genes do not enrich to any items based on this analysis. The top10 cell cycle genes are listed and of which *CCNA1* is the greater changed gene (Fig. 5B).

To confirm the RNA sequencing result, flow cytometry was performed to examine the cell cycle by monitored the DNA content in HUVECs which treated with 1,6-HD. Flow cytometry analysis reveals a decrease S phase fraction by 1,6-HD with a dose-dependent manner (Fig. 5C and D). The fraction of G1 phase increased, but no significant change of G2/M phase fraction was found (Fig. 5E and F). This data suggested1,6-HD hinders G1/S transition in HUVECs, and the effect of 1,6-HD on cell proliferation in HUVECs is likely due to an impairment of G1/S transition.

### Downregulation of cyclin A1 expression in the treatment of 1,6-hexanediol

**Fig. 6:**
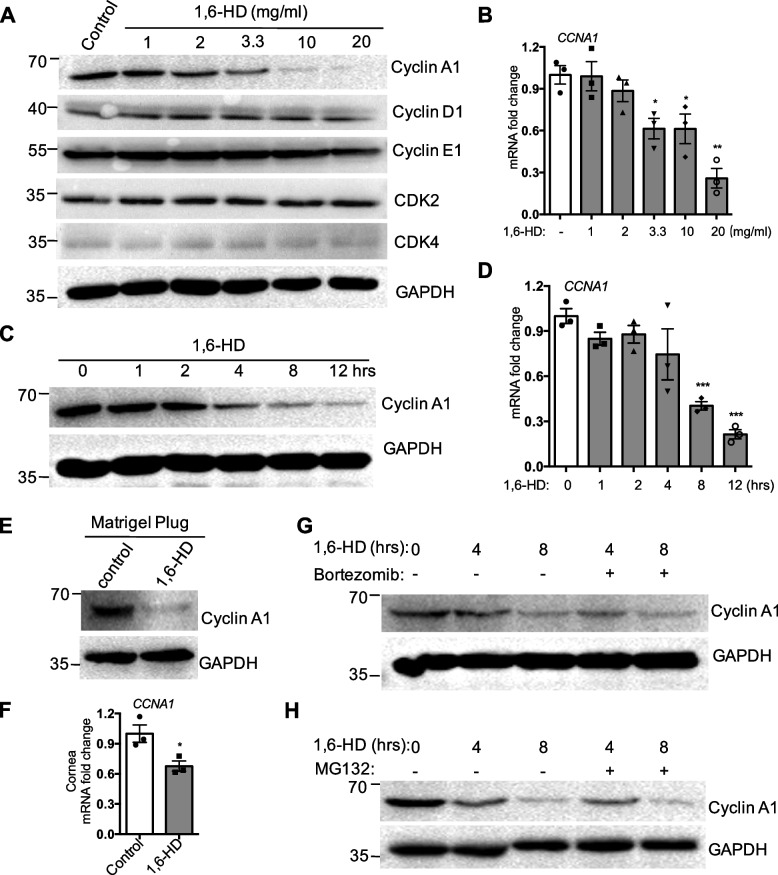
1,6-Hexanediol suppresses the expression of cyclin A1. **A** Western blot analysis of the G1/S transit-related genes in HUVECs treated with or without 1,6-HD. GAPDH was used as the loading control. **B** The mRNA expression of *CCNA1* at different concentrations of 1,6-HD treatment was determined by qRT-PCR. **C** HUVECs were treated with 1,6-HD at indicated time points. Cells were collected and the expression of cyclin A1 was analyzed by western blot. **D** In the parallel experiment, the mRNA expression of *CCNA1* was determined by qRT-PCR. **E** The expression of cyclin A1 was examined in Matrigel plugs containing 1,6-HD by Western blot. **F** The mRNA expression of CCNA1 in cornea was determined by qRT-PCR. **G**, **H** HUVECs were treated with bortezomib or MG132 for 1 h and then treated by 1,6-HD at indicated times. The expression of cyclin A1 was analyzed by Western blot. GAPDH was used as the loading control. Mean viability was shown and standard error of the mean presented the standard deviations of triplicate samples. *p < 0.05, **p < 0.01, ***p < 0.001

Since 1,6-HD results in endothelial cell cycle arrest, we sought to further examine the G1/S transition-related cell cycle genes in endothelial cells treated with 1,6-HD. Immunoblot analysis showed that the expression of cyclin A1 is greatly reduced upon 1,6-HD treatment with dose dependent (Fig. 6A), which further verified the RNA sequencing result. However, cyclin D1, cyclin E1, CDK2, and CDK4 have no significant change in the exposure of 1,6-HD (Fig. 6A). The mRNA expression is in line with protein level of these genes (Fig. 6B, Fig. S4A-D). This specific impact of 1,6-HD on *CCNA1* is further confirmed in Matrigel plugs and corneas (Fig. 6E and F, S5A). What is more, 2,5-HD does not affect the expression of cyclin A1 (Fig. S5B). We then treated HUVECs by 1,6-HD at a different time course to determinate the dynamics. 1,6-HD markedly attenuates the protein and mRNA level of cyclin A1 as early as 4 h (Fig. 6C and D). These results suggest that 1,6-HD treatment inhibits cyclin A1 expression mainly at transcriptional level. Hence, we further studied 1,6-HD regulates cyclin A1 majorly in protein level or transcriptional level. To further confirm this indication, we treated cells with proteasome inhibitors MG132 and bortezomib along with 1,6-HD. As expected, MG132 or bortezomib treatment cannot rescue the inhibitory effect of 1,6-HD on cyclin A1 expression (Fig. 6G and H). It indicates that protein degradation plays minimal role in 1,6-HD-induced *CCNA1* downregulation.

### Rescue effect of cyclin A1 on network formation upon 1,6-hexanediol treatment

**Fig. 7:**
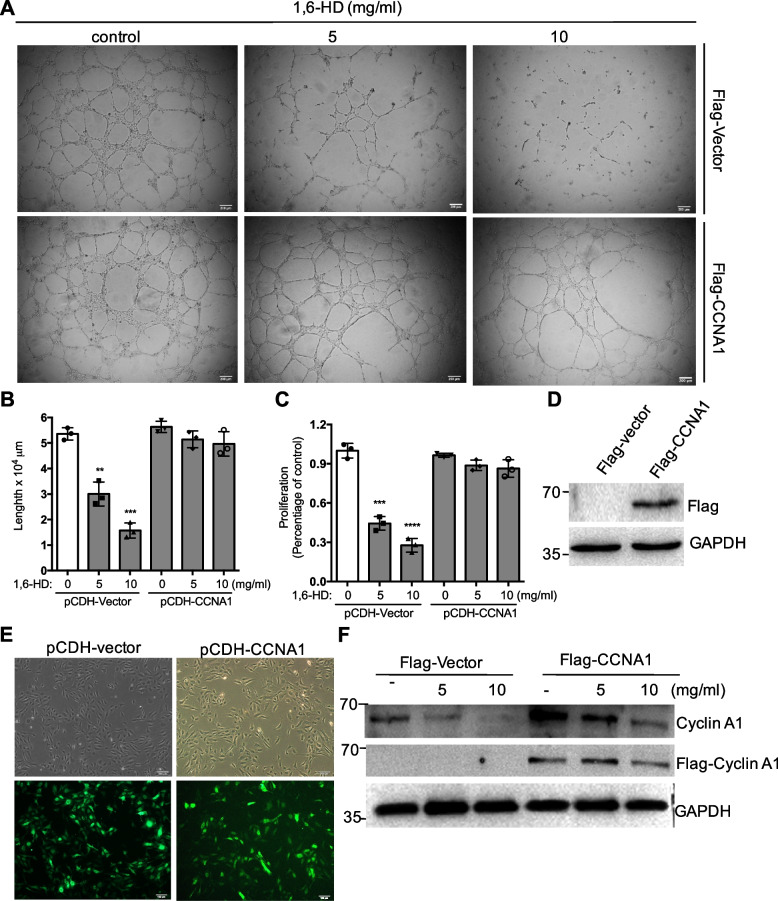
1,6-Hexanediol suppresses endothelial function through cyclin A1. **A** HUVECs were infected with lentiviral particles encoding *CCNA*1 open reading frame. After 36 h, the infected cells were treated with indicated 1,6-HD for 24 h. Tube formation assay was carried out following. **B** Quantification analysis of A. **C** Cell proliferation was examined by CCK8 assay in HUVECs overexpressed by *CCNA1*. **D**, **E** The overexpression of Cyclin A1 was analyzed. **F** The expression of endogenous cyclin A1 and exogenous Flag-cyclin A1 was detected. Mean viability was shown and standard error of the mean presented the standard deviations of triplicate samples. **p < 0.01, ***p < 0.001, ****p < 0.0001. Scale bar: 200 μm

Our above data showed that 1,6-HD attenuates angiogenesis and endothelial cell function and 1,6-HD could downregulate the expression of cyclin A1. To determine whether the dysregulated effect of 1,6-HD in angiogenesis and endothelial function is because of inhibitory expression of cyclin A1, pCDH-CMV-MSC-EF1A lentiviral vector expressing Flag-cyclin A1 was constructed. Primary HUVECs were infected with lentiviral particles containing open reading frame of *CCNA1* for 36 h, followed by 1,6-HD treatment at indicated concentrations. Tube formation assay was further performed. As shown in Fig. 7A, the network formation of control cells is dramatically reduced by 1,6-HD. Although under the high concentration of 1,6-HD, the network formation is also decreased in the cyclin A1 overexpression cells, the inhibitory effect of 1,6-HD in HUVECs is largely rescued when overexpressed cyclin A1 (Fig. 7 A and B). In the parallel experiment, cell proliferation is also rescued by overexpressing *CCNA1* in HUVECs (Fig. 7C). Furthermore, both endogenous cyclin A1 and exogenous Flag-cyclin A1 were detected by Western blot. The expression of endogenous cyclin A1 decreases by 1,6-HD consistently. The exogenous expressed cyclin A1 was detected using Flag antibodies. 1,6-HD has no effect on 1,6-HD treatment in *CCNA1* overexpressed HUVECs (Fig. 7F). The over-expressing efficiency of cyclin A1 was showed using western blot and immunofluorescence (Fig. 7D and E). This data indicated that LLPS inhibitor 1,6-HD suppresses angiogenesis and endothelial cell function through cyclin A1.

### BRD4-mediated phase separation regulates the transcription of *CCNA1*

We further explored the mechanism of *CCNA1* expression affected by LLPS in HUVECs. Cells were fixed and immunofluorescence with antibody against cyclin A1 were performed. Confocal microscope images revealed that 1,6-HD cannot affect the distribution of cyclin A1 in HUVECs, indicating that cyclin A1 does not undergo LLPS by itself (Figure S6). Recently, several reports demonstrate critical role of LLPS in the regulation of enhancer-mediated transcription [24, 25]. By analyzing the modification of H3K27ac around *CCNA1* gene from available ChIP-sequencing data in HUVECs, we excitingly found two DNA regions that are enriched with H3K27ac in HUVECs (Figure S7A). It suggests that there might be enhancer regulating the expression of *CCNA1*. We then used BRD4 (a key component for enhancer function) inhibitors JQ1 and I-BET to treat HUVECs. The expression of *CCNA1* is significantly downregulated (Figure S7B), further suggesting that enhancer controls the expression of *CCNA1*.

BRD4 is a key component for enhancers and have been proved to form phase separation condensates [24, 25]. We firstly determined whether BRD4 can form phase separation in HUVECs. The confocal images showed that BRD4 forms phase separated puncta in the nuclei of HUVECs which can be disrupted by 1,6-HD but not 2,5-HD (Figure S8). Next, we employed BET degrader dBET6 to decrease the levels of BRD4. The expression of BRD4 is greatly reduced at the concentration of 3 μM dBET6 (Figure S9A). When BRD4 is inhibited successfully, the expression of *CCNA1* fails to be further reduced in the presence of 1,6-HD (Figure S9B&C). Therefore, these data suggested that 1,6-HD suppresses the expression of *CCNA1* very likely by inhibition of BRD4 phase separation in HUVECs.

## Discussion

In this study, we showed that suppression of LLPS impairs angiogenesis by cyclin A1 medicated-EC function. 1,6-HD diminishes neovascularization in subcutaneous Matrigel plugs, impairs corneal angiogenesis response in murine corneal micropocket model, and decreases microvessel outgrowth of the aortic rings (Figs. 1 and 2). Moreover, 1,6-HD inhibits endothelial cell network formation, migration, cell growth, and proliferation (Figs. 3A and 4). Mechanically, we revealed that 1,6-HD specifically attenuates transcriptional expression of cyclin A1 and impairs endothelial cell G1/S transition (Figs. 5 and 6). Overexpression of cyclin A1 in endothelial cells could rescue dysregulated tube formation by 1,6-HD (Fig. 7). More importantly, we demonstrated that LLPS specific inhibitor 1,6-HD but not 2,5-HD diminishes neovascular formation in Matrigel plug and cornea, microvessel outgrowth of aortic ring, tube formation, proliferation of endothelial cell, and the expression of cyclin A1 (Figs. 2A, D, 3B, 4D, 6E, S1, S2D). Our study connects the new concept LLPS with endothelial cell function and angiogenesis and provides experimental evidence to show that LLPS might play a critical role in angiogenesis.

LLPS is a rapid growing biological concept which gives us new insights into understating cellular processes. Recent evidence is accumulating that LLPS contributes to gene expression, cell cycle, signaling transduction, and transcriptional regulation [13–15]. It is also critical to nuclear assemblies and cytoplasmic structures [12, 23, 26]. In addition, it has been proposed to be implicated in pathological conditions including inclusion body myopathy (IBM) and amyotrophic lateral sclerosis (ALS), as well as viral infections and many forms of cancer [19, 27, 28]. As emerging evidences is increasing regarding the link between LLPS and the processes of physiology and pathology, we are trying to bring more insights into the function of LLPS in this study. Interestingly, our data suggests that LLPS might have a fundamental role in angiogenesis although the detailed molecular mechanisms such as which protein undergoes LLPS and how it specifically control the expression of cyclin A1 need to be further explored.

1,6-HD interferes with week hydrophobic interactions between protein-RNA or protein–protein and is widely used as an inhibitor of LLPS [29]. It was firstly found to inhibit the hydrophobic interactions between nucleoporins in nuclear pore complexes (NPC) [30]. Later, 1,6-HD was found to disturb weak hydrophobic interactions between RNA-binding proteins in P granules [31]. Since then, 1,6-HD is widely used to examine the liquid droplet structures which are caused by LLPS. In this study, we used 1,6-HD to study the possible role of LLPS in endothelial cell functions and angiogenesis. We, interestingly, found that 1,6-HD inhibits endothelial cell network formation, migration, cell growth and cell cycle (Figs. 3, 4, and 5). In vivo studies support that 1,6-HD decreases angiogenesis in Matrigel plugs assay and murine corneal micropocket assay (Figs. 1 and 2). What is more, we used 2,5-hexanediol (2,5-HD), also an aliphatic alcohol, which has no effect on LLPS in living cells [23]. Notably, 1,6-HD but not 2,5-HD inhibits microvessel outgrowth of aortic ring, tube formation, and proliferation of endothelial cell (Figs. 2, 3, and 4). This further indicates that LLPS might play a role in angiogenesis and endothelial function.

It has been reported that 1,6-HD is cytotoxic to HeLa cells [21]. We also found the cytotoxic effect of 1,6-HD in several other cell lines. However, a recent study reported that there is no significant cytotoxic effect of 1,6-HD on EA.hy926 cells [22]. EA.hy926 is hybrid human endothelial cell line which was established by fusing human primary umbilical vein cells with A549 under exposure to polyethylene glycol (PEG). In this study, we used both primary HUVECs and immortalized HUVECs in the presence of several doses of 1,6-HD with different time points. Consistent with the recently published data, our result suggests that endothelial cells are relative resistant to the exposure of 1,6-HD in the low doses (Figure S2 and S3). Therefore, the cytotoxic effects of 1,6-HD in different cell type might be different and tumor cells might be more sensitive to it comparing non-cancerous cells as our previous publication [32].

Cyclin A1 was firstly cloned in murine testis which binds to both CDK1 and CDK2 [33] and then observed high expression in testis and several leukemic cell lines as well as expression in brain and somatic cell lines in humans [34]. Thus, cyclin A1 plays roles not only in the meiotic cell cycle but also in the mitotic cell cycle [35]. Cyclin A1 was also detected to express in endothelial cells, indicating have a function in endothelial cells. Several studies have shown that cyclin A1 plays a role in endothelial cell growth, cell cycle progression, and senescence [36, 37]. It also has been reported that cyclin A1 is one of a downstream molecular of heme oxygenase-1-mediated angiogenic responses [38]. Our data showed that LLPS inhibitor 1,6-HD arrests cell growth at the transition of G1/S in the cell cycle by cyclin A1. Overexpression of cyclin A1 in HUVECs could rescue the inhibitory effect of 1,6-HD on tube formation (Fig. 7*)*. It worth to notice that the expression of G1/S transition-related cell cycle genes, such as CDK2, CDK4, cyclin D1, and cyclin E1, has no big change in the present of 1,6-HD (Fig. 6A, Figure S2), indicating that 1,6-HD specifically affected the expression of cyclin A1. Our further mechanistic studies suggest that the decreases of cyclin A1 expression by 1,6-HD treatment is due to the inhibition of cyclin A1 transcription (Fig. 6G, H). It has been reported that 1,6-HD suppresses HSF1-mediated transcription in budding yeast [39]. In mammal cells, Sabari et al. found that 1,6-HD inhibits the condensates formation of coactivators at super-enhancers and then controls the transcriptional expression of genes [25], although out data indicated that 1,6-HD impaired the expression of *CCNA1* by inhibition of phase separation of BRD4. However, the detailed mechanism regarding how LLPS specifically regulates CCNA1 expression in EC is unaddressed, which deserves to be further determined in the future study. What is more, our data indicated that 1,6-HD limits angiogenesis and endothelial cell function via cyclin A1. Other possible molecular(s) regulated by 1,6-HD in endothelial cell also need to be further investigated.

In summary, we showed that suppression of LLPS impairs angiogenesis by cyclin A1 medicated-EC function. LLPS might play a critical role in angiogenesis. To our knowledge, this is the first time to use 1,6-HD to study the potential function of LLPS in angiogenesis. Although the underlying molecular mechanism regarding how LLPS inhibitor specifically regulates the expression of cyclin A1in EC is unaddressed, it is important to realize that the new concept LLPS is very likely play critical role in angiogenesis. LLPS may be a novel therapeutic target for the treatment of angiogenesis-related diseases.

## Conclusions

In this study, our data demonstrated that LLPS inhibitor 1,6-hexanediol dramatically decreases neovascularization by specific suppression of cyclin A1-mediated endothelial function, implying that the new concept LLPS is very likely play critical role in angiogenesis. LLPS could be a novel promising therapeutic target for the treatment of angiogenesis-related diseases.

## Methods

### Mice

C57BL/6 male mice 6 to 10-week-old were purchased from Beijing Vital River Laboratory Animal Technology Co., Ltd, and housed in the animal facility of Nantong University. The mice were kept on a 12 h light/12 h dark cycle at 22–23 °C. Mice were euthanized by CO_2_ inhalation or cervical dislocation.

### Reagents and chemicals

M199 medium was purchased from Hyclone (SH30253.01). ECM was purchased from Sciencell (1001). Fetal bovine serum was purchased from Gibco (10099141). DMSO was purchased from sigma (D2650). Trizol Reagent was purchased from TAKARA (9108). Protein marker (P0076) and SDS-PAGE (P0690) were purchased from Beyotime. PVDF membrane was purchased from BBI (c62-393–0100). BSA kit (C102301-0002) and DEPC water (B501005-0500) were purchased from Sangon. The Matrigel matrix was purchased from CORNING (356234). CCK-8 kit was purchased from Vazyme (A311-01/02). Flow cytometry kit was purchased from Beyotime (C1062S). Collagenase I was purchased from Maokangbio (MX1001-1000MG). MG132 and bortezomib were purchased from AbMole (M1902 and M1686). 1,6-HD and 2,5-HD were purchased from Sigma-Aldrich (240117 and H11904).

### Aortic ring assay

Male mice (8 weeks old) were sacrificed by cervical dislocation. Thoracic aorta was then isolated and transferred into a dish with sterilized 1 × PBS buffer. After removing the fibroadipose tissue, arteries were sectioned into 1-mm-long cross-sections and placed on the collagen-coated wells with corresponding concentration of 1,6-HD or 2,5-HD. Micrographs were taken after incubation at 37 °C for 2 or 3 days.

### In vivo* Matrigel angiogenesis assay*

Male mice (7–8 weeks old) were anesthetized with oxygen and 2–3% isoflurane. A mixture containing of Matrigel (Corning, USA) with corresponding concentration of 1,6-HD, 2,5-HD, or vehicle was subcutaneously injected into the dorsal surface of mice under anesthesia. Two Matrigel plugs were implanted in each mouse. One was for 1,6-HD or 2,5-HD treatment group; another was for control group. Mice were sacrificed by CO_2_ inhalation 5 days later. The Matrigel plugs were retrieved and fixed with 4% PFA for further analysis. Hematoxylin and eosin (H&E) staining, CD31 (Abcam, ab28364), and VE-cadherin (Santa Cruz, sc-9989) immunohistochemistry were carried out.

### Murine corneal micropocket assay

Male mice (8–9 weeks old) were anesthetized with an intraperitoneal injection of a combination of ketamine 100 mg/kg and xylazine 10 mg/kg. The eye is further anesthetized with few drop of 0.5% proparacaine. Then, an incision into the cornea approximately 1 mm from the limbus was made. The incision should be 1–1.5 mm in length. A pellet with 1,6-HD or 2,5-HD was implanted into the micropocket in one eye. A pellet without 1,6-HD or 2,5-HD was implanted into the micropocket in another eye. Pellets (with /without 1,6-HD or 2,5-HD) were prepared ahead. Seven days after pellet implantation, observations of the implants were made by a slip lamp microscope on anaesthetized mice with isoflurane. Immunofluorescence of CD31 (BD Bioscience, 553,369) and FITC-lectin (Vector labs, FL1101-5) was further performed.

### Primary HUVECs isolation

Umbilical cord was obtained from a healthy donor. Human umbilical vein endothelial cells were then isolated by digestion with 0.1% collagenase I at 37 °C for 15 min in incubator. The solution was collected and centrifuged at 1000 × g for 5 min. The pellets were resuspended in M199 medium. Then cells were cultured in M199 full medium. The study on the protocol of HUVECs isolation was in compliance with the Declaration of Helsinki.

### RNA extraction and quantitative RT-PCR

Total RNA was extracted with Trizol reagent (TAKARA, Japan). The quality and concentration were tested by the Nanodrop. Then, approximately 1 μg of RNA was reverse transcribed with HiScript II-RT SuperMix for qPCR (+ gDNAwiper) (Vazyme, Nanjing, China). Quantitative PCR was carried out in triplicate with AceQ Universal SYBR qPCR Master Mix (Vazyme, Nanjing, China) on a StepOne Plus Real-Time PCR System (Applied Biosystems. Foster City, CA). The 18S served as the housekeeping control. Relative mRNA expression levels were calculated using the 2^−ΔΔCt^ method and normalized to the internal reference gene 18S.

The primers used are listed as follows: Human cyclinA1: Forward: GAGGTCCCGATGCTTGTCAG, Reverse: GTTAGCAGCCCTAGCACTGTC. Human cyclinD1: Forward: CAATGACCCCGCACGATTTC, Reverse: CATGGAGGGCGGATTGGAA. Human cyclinE1: Forward: AAGGAGCGGGACACCATGA, Reverse: ACGGTCACGTTTGCCTTCC. Human CDK2: Forward: GTACCTCCCCTGGATGAAGAT, Reverse: CGAAATCCGCTTGTTAGGGTC. Human CDK4: Forward: TCAGCACAGTTCGTGAGGTG, Reverse: GTCCATCAGCCGGACAACAT. Mouse cyclinA1: Forward: TGATGCTTGTCAAATGCTCAGC, Reverse: AGGTCCTCCTGTACTGCTCAT. Mouse CD31: Forward: CTGCCAGTCCGAAAATGGAAC, Reverse: CTTCATCCACCGGGGCTATC. Mouse VE-cadherin: Forward: CACTGCTTTGGGAGCCTTC, Reverse: GGGGCAGCGATTCATTTTTCT. 18S: Forward: GAACGAGCTGGCATGCTA, Reverse: CACGTGAGCCAGTCAGTGTA.

### Protein extraction and western blot

All the cells were lysed in radioimmunoprecipitation (RIPA) buffer supplemented with 1% proteinase inhibitors (Beyotime, Shanghai, China) on ice for 30 min. Protein were extracted by centrifuged for 15 min at 4 °C and quantitatively analyzed using a BCA kit (Sangon, Shanghai, China). An equal amount of protein from each sample was separated by 10% SDS PAGE and then transferred to the PVDF membrane (BBI, USA). The membranes were incubated overnight at 4 °C with indicated primary antibodies and following incubated with the corresponding secondary antibodies. Anti-cyclinA (sc-271682), anti-cyclin D (sc-8396), anti-cyclin E (sc-377100), anti-CDK2 (sc-6248), and anti-CDK4 (sc-23896) antibodies were purchased from Santa Cruz; anti-GAPDH (AF1186) were purchased from Beyotime Biotechnology. The anti-mouse IgG secondary antibodies were purchased from Sino biological (SSA007), and anti-rabbit IgG secondary antibodies were purchased from Bioworld Technology (BS13278).

### RNA sequencing

HUVECs (ATCC, CRL-1730) were treated by 1 mg/ml and 3.3 mg/ml 1,6-HD for 16 h. RNA sequencing was conducted with the help of Beijing Genomic Institution (BGI). Briefly, mRNA molecules were purified from total RNA using oligo(dT)-attached magnetic beads. After mRNA fragment, cDNA synthesis, end repair, and add A and adaptor ligation, cDNA fragments were amplified and PCR product were purified with Ampure XP Beads (AGENCOURT). Then, library was validated on the Agilent Technologies 2100 bioanalyzer. After circularization, the single strand circle DNA (ssCir DNA) were formatted as the final library. Then, the library was amplified with phi29 to make DNA nanoball (DNB), which were load into patterned nanoarray, and 150 pair end bases reads were generated in the way of combinatorial Probe-anchor Synthesis (cPAS). After quality control, clean and high-quality data obtained and stored in FASTQ format. The clean reads were mapped to the reference genome using HISAT2 (v2.0.4) Bowtie2 (v2.2.5) was applied to align the clean reads to the reference coding gene set, and then expression level of gene was calculated by RSEM (v1.2.12). The most downregulated genes were analyzed by online gene set enrichment analysis (GSEA) program.

### Endothelial cell proliferation assay, migration assay, and network formation assay

Cell proliferation was assessed by CCK-8 kit (Vazyme, Nanjing, China) following the instructions of manufacturer. Briefly, HUVECs were seeded into 96-well plates at a density of 2 × 10^3^ cells per well for 24 h, and then the medium was replaced by 200 μl complete medium with indicated concentration of 1,6-HD or 2,5-HD. After 24 h, 10 μl of CCK-8 solution was added into each well and incubated at 37 °C for 2 h. Finally, cell optical density was determined at 450 nm through a microplate reader (Thermo Fisher Scientific). In order to detect the ability of cell migration, wound healing assay was used. Approximately 4 × 10^5^ cells/well were seeded in six-well plates. After the cells reached 70% confluency, wounds were created using a 10-μl pipette tip. Cells were treated with corresponding concentration of 1,6-HD. Then, the cells were incubated for at 37° C, 5% CO_2_. Images were taken 0, 24, 36, and 48 h later. For network formation assay, approximately 4 × 10^5^ cells/well were seeded in six-well plates with ECM overnight, and then starved using DMEM for 12 h. After starvation, replacing DMEM with ECM including different concentrations of 1,6-HD or 2,5-HD. A 96-well plate was coated with 50 μl Matrigel (Corning) and kept at 37 °C for 30 min. A total of 2 × 10^4^ HUVECs suspended in 100 μl conditioned medium were applied to the precoated 96-well plate. Micrographs were taken after incubation at 37 °C for another 6 h.

### Flow cytometry analysis

Approximately 3 × 10^5^ HUVECs/well were seeded in six-well plates and then treated with different concentrations of 1,6-HD for 36 h, and using trypsin (without EDTA) to collect cells. The cells were fixed with 70% ethanol overnight at 4 °C in a refrigerator. Then, the cells were washed gently with PBS twice and incubated for 15–20 min with PI in dark. The samples were analyzed using a BD flow cytometer (BD Biosciences).

### Statistical analysis

All the data were analyzed using Prism. The unpaired two-side *t* test was used to determine the differences between two groups, and one-way ANOVA was used for multiple-group comparison. *P* < 0.05 was considered statistically significant. *P < 0.05, **P < 0.01, ***P < 0.001, and ****P < 0.0001.

## Supplementary Information

**Additional file 1: **Figure S1-S9. **Fig. S1** 2,5-hexanediol has no effect on blood vessel formation in Matrigel plugs. (A) Matrigel mixed with/without 2,5-HD (20 mg/ml) was injected into C57BL/6 mice subcutaneously. The gross morphology of Matrigel plugs was shown after five days’ injection. (B-D) Matrigel plugs were fixed by PFA. After section, the hematoxylin and eosin (H&E) staining, CD31 and VE-cadherin immunohistochemistry of Matrigel plugs was performed and shown. Scale bar: 50 μm. **Fig. S2** Morphology of endothelial cell after 1,6-hexanediol treatment. HUVECs were treated with indicated concentrations of 1,6-HD. Images were taken on day 1, day 2 and day 3, respectively. Scale bar: 100 μm. **Fig. S3** Effect of 1,6-hexanediol on endothelial cell viability. Cell viability was monitored by propidium iodide (PI) which can stain the dead cells with red. Scale bar: 100 μm. **Fig. S4** 1,6-HD has no effect on other G1/S transition-related genes. (A-D) HUVECs were treated by 1,6-HD at indicated concentrations for 16 h. Total RNA was extracted. The mRNA expressions of *CCND1, CCNE1, CDK2 and CDK4* were determined by qRT-PCR. **Fig. S5** 1,6-HD but no 2,5-HD affects Cyclin A1 expression. (A) Matrigel plug assay was performed. The mRNA expression of *CCNA1* was examined in plugs with or without 1,6-HD by qRT-PCR. (B) HUVECs were treated by 2,5-HD at different time points. Protein were extracted. The expression of Cyclin A1 was analyzed by western blot. Mean viability was shown and standard error of the mean presented the standard deviations of triplicate samples. ***** *p* < 0.00001. **Fig. S6** 1,6-HD cannot affect the distribution of Cyclin A1 in HUVECs. (A) HUVECs were treated with 1,6-HD for 2 h. Immunofluorescence of Cyclin A1 was performed. Confocal images were shown. (B) Magnification of the areas indicated in A. Scale bar: 10 μm. **Fig. S7** Inhibiting super-enhancer downregulates the expression of CCNA1. (A) The published ChIP sequencing data were extracted and analyzed the H3K27ac levels in HUVECs around CCNA1. (B) HUVECs were treated with or without JQ1 and I-BET. The expression of CCNA1 was determined by qRT-PCR. Mean viability was shown and standard error of the mean presented the standard deviations of triplicate samples. *** *p* < 0.001, **** *p* < 0.0001. **Fig. S8** 1,6-HD but not 2,5-HD disrupts BRD4 accumulation in HUVECs. (A) HUVECs were treated by 1,6-HD and 2,5-HD with indicated time. Cells were fixed and immunofluorescent staining of BRD4 were carried out. Images were obtained by confocal microscope. (B) Magnification of the areas indicated in A. Scale bar: 10 μm. **Fig. S9** 1,6-HD fails to further reduced CCNA1 after reduction of BRD4 protein. (A) HUVECs were treated with dBET6 for 6 h. The expression of BRD4 was analyzed by western blot. (B, C) HUVECs were treated with 3 μM dBET6, then 10 mg/ml 1,6-HD were further treated. The expression of CCNA1 was examined by qRT-PCR and Western blot. Mean viability was shown and standard error of the mean presented the standard deviations of triplicate samples. ** *p* < 0.01.

## Data Availability

All data generated or analyzed during this study are included in this published article, its supplementary information files, and publicly available repositories. The RNA sequence datasets generated during this study are also available at China National GeneBank DataBase (CNGBdb: CNP0004149).

## Abbreviations

LLPS: Liquid-liquid phase separation
1,6-HD: 1,6-Hexanediol
2,5-HD: 2,5-Hexanediol
EC: Endothelial cells
VEGF: Vascular endothelial growth factor
IHC: Immunohistochemistry
HUVECs: Human umbilical vein endothelial cells
IBM: Inclusion body myopathy
ALS: Amyotrophic lateral sclerosis
NPC: Nuclear pore complexes
PEG: Polyethylene glycol
H&E: Hematoxylin and eosin
RIPA: Radioimmunoprecipitation
