# Supplementary material for: 1,6-Hexanediol regulates angiogenesis via suppression of cyclin A1-mediated endothelial function
Source: BMC Biol. 2023 Apr 7;21:75. doi: 10.1186/s12915-023-01580-8 (PMC10080975; doi:10.1186/s12915-023-01580-8)
Supplement: Supplementary file 1 — Additional file 1: Figure S1-S9. Fig. S1 2,5-hexanediol has no effect on blood vessel formation in Matrigel plugs. (A) Matrigel mixed with/without 2,5-HD (20 mg/ml) was injected into C57BL/6 mice subcutaneously. The gross morphology of Matrigel plugs was shown after five days’ injection. (B-D) Matrigel plugs were fixed by PFA. After section, the hematoxylin and eosin (H&E) staining, CD31 and VE-cadherin immunohistochemistry of Matrigel plugs was performed and shown. Scale bar: 50 μm. Fig. S2 Morphology of endothelial cell after 1,6-hexanediol treatment. HUVECs were treated with indicated concentrations of 1,6-HD. Images were taken on day 1, day 2 and day 3, respectively. Scale bar: 100 μm. Fig. S3 Effect of 1,6-hexanediol on endothelial cell viability. Cell viability was monitored by propidium iodide (PI) which can stain the dead cells with red. Scale bar: 100 μm. Fig. S4 1,6-HD has no effect on other G1/S transition-related genes. (A-D) HUVECs were treated by 1,6-HD at indicated concentrations for 16 h. Total RNA was extracted. The mRNA expressions of CCND1, CCNE1, CDK2 and CDK4 were determined by qRT-PCR. Fig. S5 1,6-HD but no 2,5-HD affects Cyclin A1 expression. (A) Matrigel plug assay was performed. The mRNA expression of CCNA1 was examined in plugs with or without 1,6-HD by qRT-PCR. (B) HUVECs were treated by 2,5-HD at different time points. Protein were extracted. The expression of Cyclin A1 was analyzed by western blot. Mean viability was shown and standard error of the mean presented the standard deviations of triplicate samples. ***** p < 0.00001. Fig. S6 1,6-HD cannot affect the distribution of Cyclin A1 in HUVECs. (A) HUVECs were treated with 1,6-HD for 2 h. Immunofluorescence of Cyclin A1 was performed. Confocal images were shown. (B) Magnification of the areas indicated in A. Scale bar: 10 μm. Fig. S7 Inhibiting super-enhancer downregulates the expression of CCNA1. (A) The published ChIP sequencing data were extracted and analyzed the H3K27ac levels in [file 12915_2023_1580_MOESM1_ESM.docx]

**Additional File1: Figure S1-S9**

**Fig S1 2,5-hexanediol has no effect on blood vessel formation in Matrigel plugs.**

**(A)** Matrigel mixed with/without 2,5-HD (20mg/ml) was injected into C57BL/6 mice subcutaneously. The gross morphology of Matrigel plugs was shown after five days’ injection. (**B-D)** Matrigel plugs were fixed by PFA. After section, the hematoxylin and eosin (H&E) staining, CD31 and VE-cadherin immunohistochemistry of Matrigel plugs was performed and shown. Scale bar: 50 μm.

**Fig S2 Morphology of endothelial cell after 1,6-hexanediol treatment.**

HUVECs were treated with indicated concentrations of 1,6-HD. Images were taken on day 1, day 2 and day 3, respectively. Scale bar: 100 μm.

**Fig S3 Effect of 1,6-hexanediol on endothelial cell viability.**

Cell viability was monitored by propidium iodide (PI) which can stain the dead cells with red. Scale bar: 100 μm.

**Fig S4 1,6-HD has no effect on other G1/S transition-related genes.**

**(A-D)** HUVECs were treated by 1,6-HD at indicated concentrations for 16 hours. Total RNA was extracted. The mRNA expressions of *CCND1, CCNE1, CDK2 and CDK4* were determined by qRT-PCR.

**Fig S5 1,6-HD but no 2,5-HD affects Cyclin A1 expression**

**(A)** Matrigel plug assay was performed. The mRNA expression of *CCNA1* was examined in plugs with or without 1,6-HD by qRT-PCR. (**B)** HUVECs were treated by 2,5-HD at different time points. Protein were extracted. The expression of Cyclin A1 was analyzed by western blot. Mean viability was shown and standard error of the mean presented the standard deviations of triplicate samples. ***** *p* < 0.00001

**Fig S6 1,6-HD cannot affect the distribution of Cyclin A1 in HUVECs.**

**(A)** HUVECs were treated with 1,6-HD for 2 hours. Immunofluorescence of Cyclin A1 was performed. Confocal images were shown. (**B)** Magnification of the areas indicated in A. Scale bar: 10 μm.

**Fig S7 Inhibiting super-enhancer downregulates the expression of CCNA1**

**(A)** The published ChIP sequencing data were extracted and analyzed the H3K27ac levels in HUVECs around CCNA1. (**B)** HUVECs were treated with or without JQ1 and I-BET. The expression of CCNA1 was determined by qRT-PCR. Mean viability was shown and standard error of the mean presented the standard deviations of triplicate samples. *** *p* < 0.001, **** *p* < 0.0001.

**Fig S8 1,6-HD but not 2,5-HD disrupts BRD4 accumulation in HUVECs.**

**(A)** HUVECs were treated by 1,6-HD and 2,5-HD with indicated time. Cells were fixed and immunofluorescent staining of BRD4 were carried out. Images were obtained by confocal microscope. (**B)** Magnification of the areas indicated in A. Scale bar: 10 μm.

**Fig S9 1,6-HD fails to further reduced CCNA1 after reduction of BRD4 protein.**

**(A)** HUVECs were treated with dBET6 for 6 hours. The expression of BRD4 was analyzed by western blot. (**B, C)** HUVECs were treated with 3μM dBET6, then 10mg/ml 1,6-HD were further treated. The expression of CCNA1 was examined by qRT-PCR and Western blot. Mean viability was shown and standard error of the mean presented the standard deviations of triplicate samples. ** *p* < 0.01.


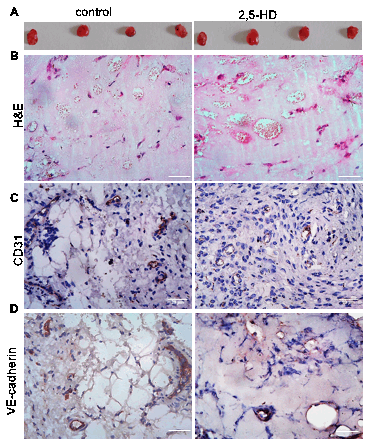


**Fig S1 2,5-hexanediol has no effect on blood vessel formation in Matrigel plugs**

**(A)** Matrigel mixed with/without 2,5-HD (20mg/ml) was injected into C57BL/6 mice subcutaneously. The gross morphology of Matrigel plugs was shown after five days’ injection. (**B-D)** Matrigel plugs were fixed by PFA. After section, the hematoxylin and eosin (H&E) staining, CD31 and VE-cadherin immunohistochemistry of Matrigel plugs was performed and shown. Scale bar: 50 μm.


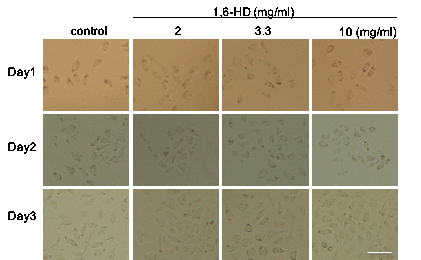


**Fig S2 Morphology of endothelial cell after 1,6-hexanediol treatment.**

HUVECs were treated with indicated concentrations of 1,6-HD. Images were taken on day 1, day 2 and day 3, respectively. Scale bar: 100 μm.


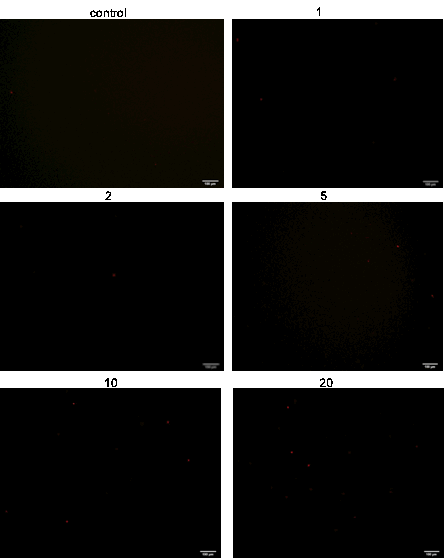


**Fig S3 Effect of 1,6-hexanediol on endothelial cell viability.**

Cell viability was monitored by propidium iodide (PI) which can stain the dead cells with red. Scale bar: 100 μm.


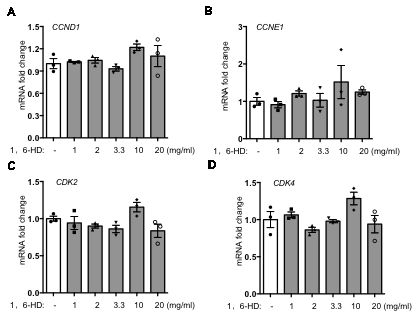


**Fig S4 1,6-HD has no effect on other G1/S transition-related genes.**

**(A-D)** HUVECs were treated by 1,6-HD at indicated concentrations for 16 hours. Total RNA was extracted. The mRNA expressions of *CCND1, CCNE1, CDK2 and CDK4* were determined by qRT-PCR.


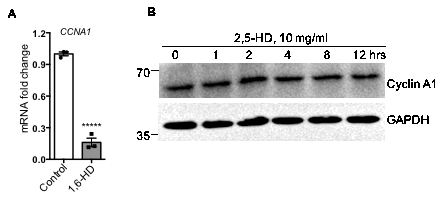


**Fig S5 1,6-HD but no 2,5-HD affects Cyclin A1 expression**

**(A)** Matrigel plug assay was performed. The mRNA expression of *CCNA1* was examined in plugs with or without 1,6-HD by qRT-PCR. (**B)** HUVECs were treated by 2,5-HD at different time points. Protein were extracted. The expression of Cyclin A1 was analyzed by western blot. Mean viability was shown and standard error of the mean presented the standard deviations of triplicate samples. ***** *p* < 0.00001


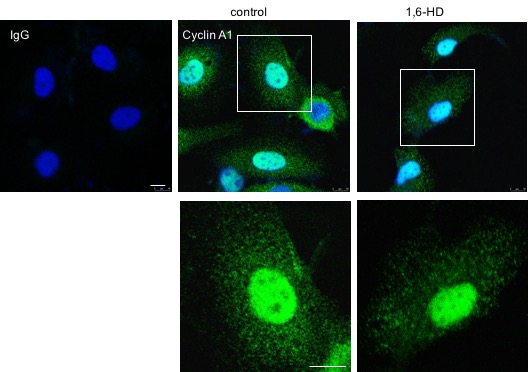


**Fig S6 1,6-HD cannot affect the distribution of Cyclin A1 in HUVECs.**

**(A)** HUVECs were treated with 1,6-HD for 2 hours. Immunofluorescence of Cyclin A1 was performed. Confocal images were shown. (**B)** Magnification of the areas indicated in A. Scale bar: 10 μm.


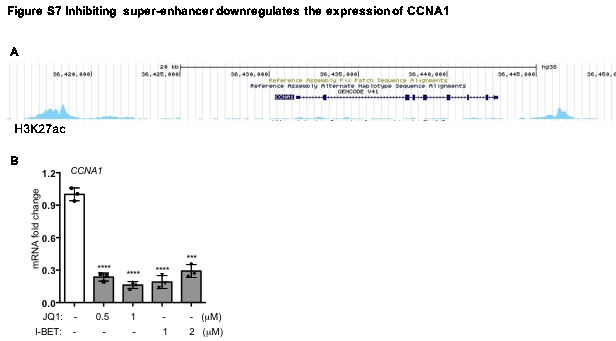


**Fig S7 Inhibiting super-enhancer downregulates the expression of CCNA1**

**(A)** The published ChIP sequencing data were extracted and analyzed the H3K27ac levels in HUVECs around CCNA1. (**B)** HUVECs were treated with or without JQ1 and I-BET. The expression of CCNA1 was determined by qRT-PCR. Mean viability was shown and standard error of the mean presented the standard deviations of triplicate samples. *** *p* < 0.001, **** *p* < 0.0001.


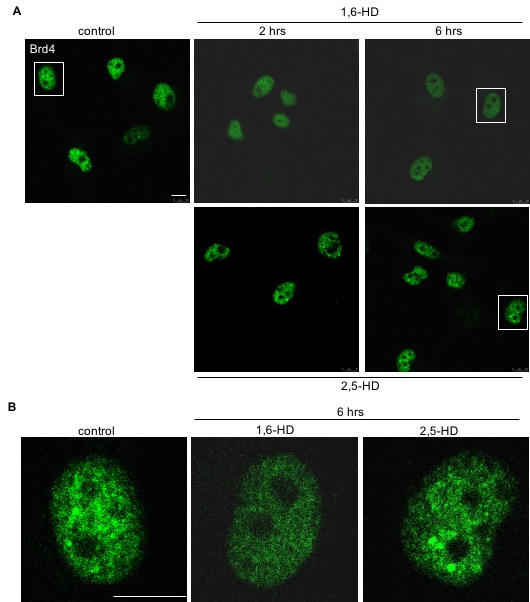


**Fig S8 1,6-HD but not 2,5-HD disrupts BRD4 accumulation in HUVECs.**

**(A)** HUVECs were treated by 1,6-HD and 2,5-HD with indicated time. Cells were fixed and immunofluorescent staining of BRD4 were carried out. Images were obtained by confocal microscope. (**B)** Magnification of the areas indicated in A. Scale bar: 10 μm.


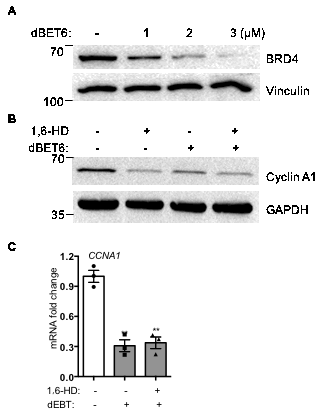


**Fig S9 1,6-HD fails to further reduced CCNA1 after reduction of BRD4 protein.**

**(A)** HUVECs were treated with dBET6 for 6 hours. The expression of BRD4 was analyzed by western blot. (**B, C)** HUVECs were treated with 3μM dBET6, then 10mg/ml 1,6-HD were further treated. The expression of CCNA1 was examined by qRT-PCR and Western blot. Mean viability was shown and standard error of the mean presented the standard deviations of triplicate samples. ** *p* < 0.01.
